# 3D computer tomography for measurement of femoral position in acl reconstruction

**DOI:** 10.1590/1413-78522015230100993

**Published:** 2015

**Authors:** Tiago Lazzaretti Fernandes, Nuno Miguel Morais Fonseca Martins, Felipe de Andrade Watai, Cyro Albuquerque, André Pedrinelli, Arnaldo José Hernandez

**Affiliations:** I University of São Paulo, Hospital das Clínicas, Institute of Orthopedics and Traumatology, Brazil, Institute of Orthopedics and Traumatology, Hospital das Clínicas, University of São Paulo, Medical School. Brazil; II IFEI University Center, Department of Mechanical Engineering, São Paulo, SP, Brazil, FEI University Center, Department of Mechanical Engineering, São Paulo, SP, Brazil

**Keywords:** Anterior cruciate ligament, Anatomy, Image processing computer-assisted, Imaging, three-dimensional

## Abstract

**Objective:**

To validate intra- and inter-class correlation coefficients of a transparent 3D-TC protocol and investigate relationships between different axial rotations.

**Methods:**

Twenty unilateral knee TCs (iSite - Philips) were evaluated by means of a transparent 3D-TC OsiriX Imaging Software (v.3.9.4), 3D MPR protocol. Mathematical model of femoral tunnel projections acquired on vertical and horizontal rotations from -20 to +20 degrees. Height (h'/H) and length (t'/T) of tunnel projections have been analyzed by the Bernard and Hertel's method.

**Statistics:**

power of study=80%, ICC, ANOVA, p<0.05 (SPSS-19). Results: Transparent 3D-TC showed high reliability of both intra-observer (h'/H=0.941; t'/T=0.928, p<0.001) and inter-observer (h'/H=0.921; t'/T=0.890, p<0.001) ICC. ACL Length (t'/T) and Height (h'/H) projections were statistically different on vertical and horizontal rotations: p=0.01 and p<0.001, respectively.

**Conclusion:**

This new transparent 3D-TC protocol is an accurate and reproducible method that can be applied for ACL femoral tunnel or footprint measurement with high ICC reliability. Level of Evidence II, Descriptive Laboratory Study.

## Introduction

Volume-rendering 3D computed tomography (CT) scan is the preferred imaging technique to evaluate osseous anatomy of the knee and anatomic femoral tunnel position after anterior cruciate ligament (ACL) reconstruction.^1^^-^ 8

**Figure 1.**
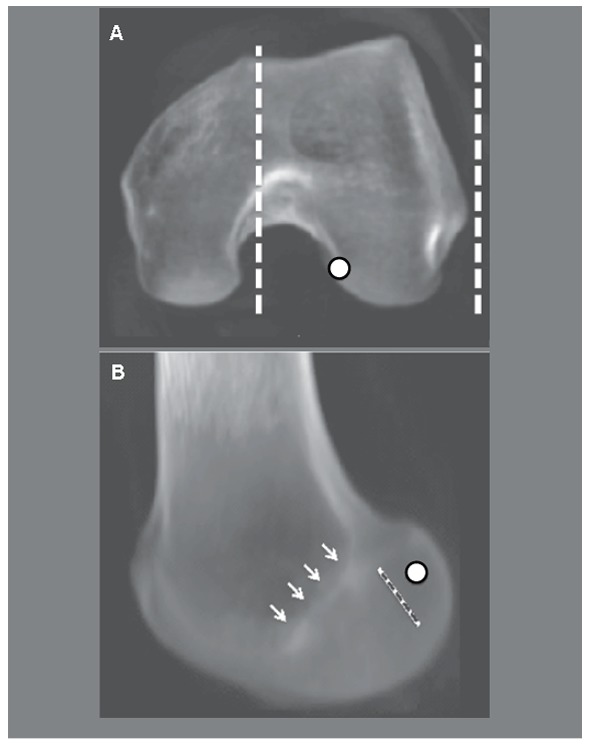
Transparent CT scan of the lateral femoral condyle. A) Dotted line: limit of the lateral femoral condyle including the intercondylar notch. B) Sagittal view of the selected lateral condyle. White arrows: Blumensaat line; dotted line: resident ridge; circle: central ACL footprint.

Clinical or biomechanical studies may show an inaccurate relationship between functional outcomes and ACL tunnel positioning if standardized and validated 3D CT scan protocols are not employed.^9^ Conventional rendered 3D CT has some disadvantages, since standard references for ACL measurement, as Blumensaat line, are not precisely analyzed.^9^ On the other hand, transparent 3D CT allows simultaneous visualization of femoral condyle margin, Blumensaat line full projection and also ACL tunnel positioning when present. 9 ^-^ 11 (Figure 1)

Here, we propose an accessible transparent 3D CT measurement protocol for central femoral footprint or ACL tunnel positioning assessment, taking account condyle femoral alignment and a standardized projection of Blumensaat line, as from a mathematical model. 

Thus, the purpose of this study was to validate intra- and inter-class correlation coefficients of a standardized new transparent 3D CT scan for femoral footprint ACL measurement with a simulation of central ACL tunnel position in different axial rotations in both vertical and horizontal axes, as it was an imaging laboratory study. 

It was hypothesized that horizontal and vertical rotations could alter the relationship between radiological landmarks (Blumensaat line and lateral condyle wall) and simulated central ACL position in Bernard *et al.* 12 method in this transparent 3D CT protocol.

## Materials and methods

We conducted an image laboratory study of 20 consecutive unilateral knees CT scans presented in our database (2012) from skeletally mature bone to 45-year-old patients (volumetric acquisition: 0.06mm - Discovery CT750 HD, 64 slice, GE). Individuals were not identified, and subjects with previous surgery or trauma about the knee were excluded from this study. The study was approved by our Institution Review Board (IRB) before data analysis has begun.

3D CT Reconstruction of Lateral Femoral Condyle: Computed tomography DICOM files were processed into commercial OsiriX(r) Imaging Software (v.3.9.4). Images were acquired including intercondylar notch and lateral condyle in a bone transparent image technique similar to radiographies available in this software (3D MPR protocol). Figure 1

**Figure 2.**
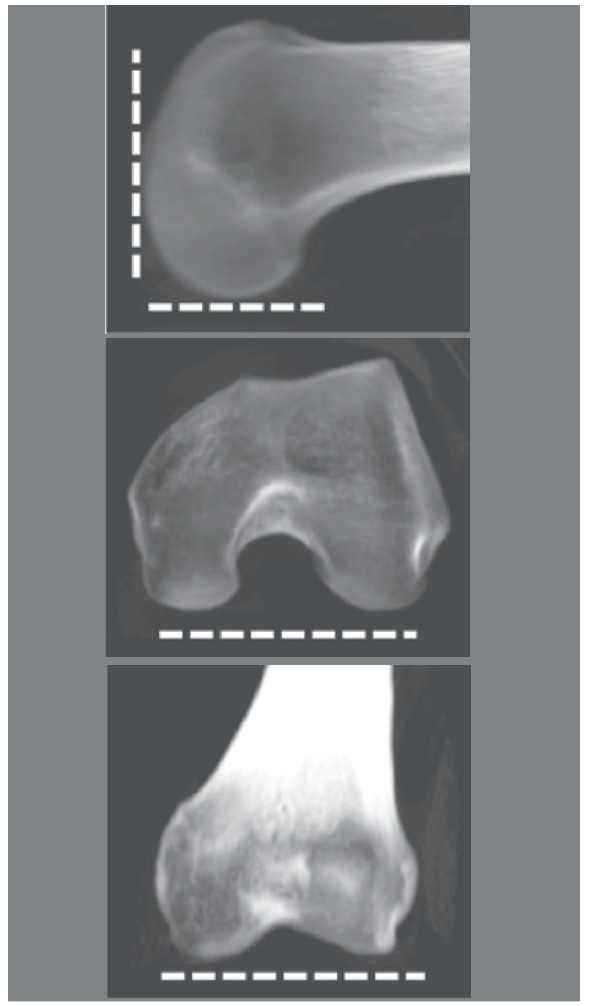
Transparent CT scan: inferior and posterior condyle wall alignment in sagittal, axial and coronal view.

The "true lateral view"9,13 was standardized by aligning posterior femoral condyle walls in sagittal and axial view and inferior walls in sagittal and coronal view. Figure 2

**Figure 3.**
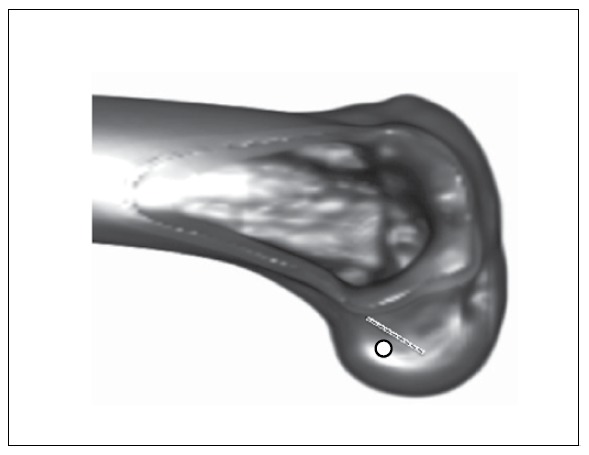
Simulated central ACL tunnel positioning (circle) in a rendered and cropped 3D CT scan. Dotted line: resident ridge. Note the selected central ACL position below resident ridge.

ACL Measurement on transparent 3D CT Scan: Each data was rendered as a 3D volume knee. Lateral femoral condyle was isolated by cropping medial condyle from this 3D rendering knee. Simulated central ACL position was determined as a central point below lateral intercondylar ridge and in the middle of ACL footprint as carefully described and presented by Kopf *et al.* 7 and other authors.^6^ Lateral bifurcate ridge was used as reference, when visible.^14^ (Figure 3)

**Figure 4.**
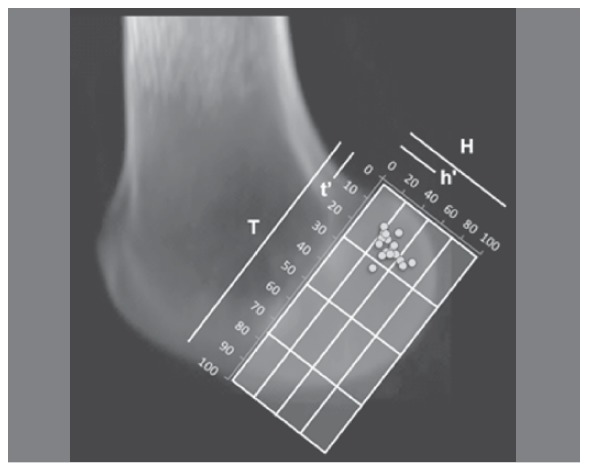
Bernard and Hertel12 quadrant method in a neutral transparent CT scan of the lateral femoral condyle. T = total condyle length, t'= central ACL percentage of T; H = total height, h' = central ACL percentage of H. Yellow dot = simulated central ACL footprint position of each subject.

The same simulated central ACL position was loaded in the transparent sagittal view, as described before, and the quadrant method of Bernard *et al.* 12 was applied in the picture obtained, as described by Lertwanich *et al.* 15 and Kai *et al.* 10 In 3D CT scans. A line connecting the most anterior and posterior edge of the intercondylar roof was the reference for Blumensaat line. The inferior border of the rectangle was a line tangent to the most distal point in the lateral condyle. The anterior and posterior edges of the lateral femoral condyle served as the other two borders to make the grid. ACL positioning was defined as a percentage of the total sagittal diameter of the lateral condyle and intercondylar notch height. Figure 4

Two independent and post graduate orthopedic surgeons familiar with OsiriX software evaluated, individually, 13 images (neutral position and rotations) in every CT scan (20) in the initial inter-observer analysis. Intra-observer analysis was repeated after 4-week interval by one of them to perform inter-observer analysis as described in literature.

**Figure 5.**
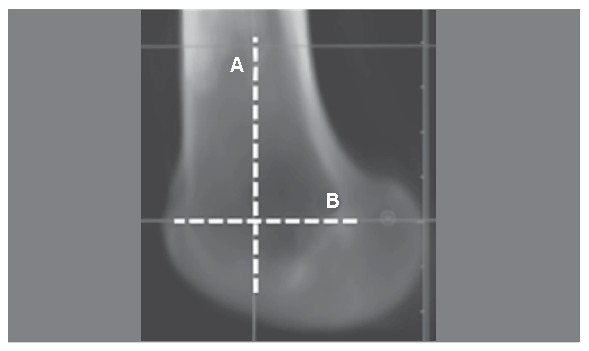
Femoral rotation axes - A) vertical axis (internal/external femoral rotation); B) horizontal axis (femoral adduction/abduction)

Vertical axis was defined as a perpendicular line to the aligned inferior femoral condyle wall and centralized in the lateral condyle in coronal and axial views. Horizontal axis was set as a perpendicular line to the posterior femoral condyle wall in the same sagittal view and in the height of lateral and medial femoral epicondyles in coronal view. (Figure 5)

**Figure 6.**
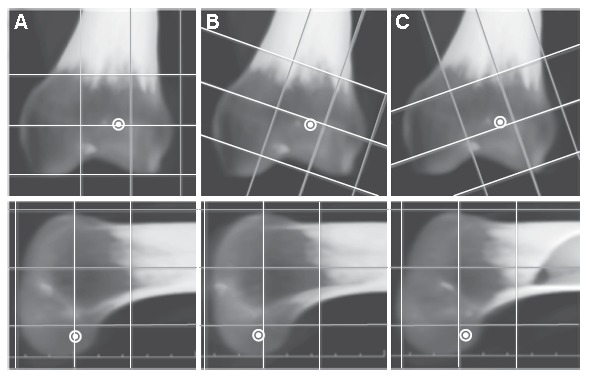
Horizontal rotations of transparent CT scan of the same knee. Note the difference in simulated positions of ACL tunnels (white target) - Column A) Sagittal view (bottom) of a correctly aligned CT scan; Column B) Sagittal view (bottom) of a abducted CT scan; Column C) Sagittal view (bottom) of a adducted CT scan.

Rotations were made on vertical and horizontal axis with -20, -10, 0, +10 and +20 degrees, respectively, using an angle tool included in the OsiriX software. (Figure 6)

## Statistics

All statistical analysis was performed with SPSS version 19.0 for Windows (SPSS Inc Chicago, Illinois). Statistical significance was set at p<0.05. The inter- and intra-observer reliability (intraclass correlation coefficient - ICC) of central femoral ACL footprint measured by Bernard *et al.* 12 quadrant method were calculated. A measurement was considered reliable if the ICC was higher than 0.80, as described in similar studies.^15^ We also used the analysis of variance (ANOVA) and Bonferroni post-hoc test for relationship between different rotations.

Sample size calculation was defined considering primary outcome as Bernard and Hertel length ratios in different rotations with p<0.05 and power of study=80%. Standard deviation between different measures was 2.58 and n=15. We pondered more five subjects due to possibility of lost data.

## Results

It was selected 20 consecutive knees CT scan from 14 men and six women, mean age of 31 years (range, 17 to 43).

There was a high reliability of Intraclass Correlation Coefficient (ICC) for both intra and inter-observer measurements related to this transparent image technique method. The intra-observer ICC of ACL length ratios over total sagittal diameter of the lateral condyle and intercondylar notch height were 0.93 and 0.94, respectively (p<0.001). Inter-observer ICC were 0.89 and 0.92, respectively (p<0.001). 

The length ratios of simulated central ACL tunnel distances (along Blumensaat line and intercondylar notch height) according to Bernard and Hertel method were 20.9% ± 3.6% (mean ± sd) and 35.9% ± 10.4% (mean ± sd), respectively. (Figure 4) 

For rotations of -20, -10, 0, +10 and +20 degrees, there were statistically significant differences on ACL length ratios on vertical and horizontal axes (ANOVA - F[6,20]= 2.23, p=0.04 and F[6,20]=7.64, p=0.001, respectively and ANOVA -

**Figure 7.**
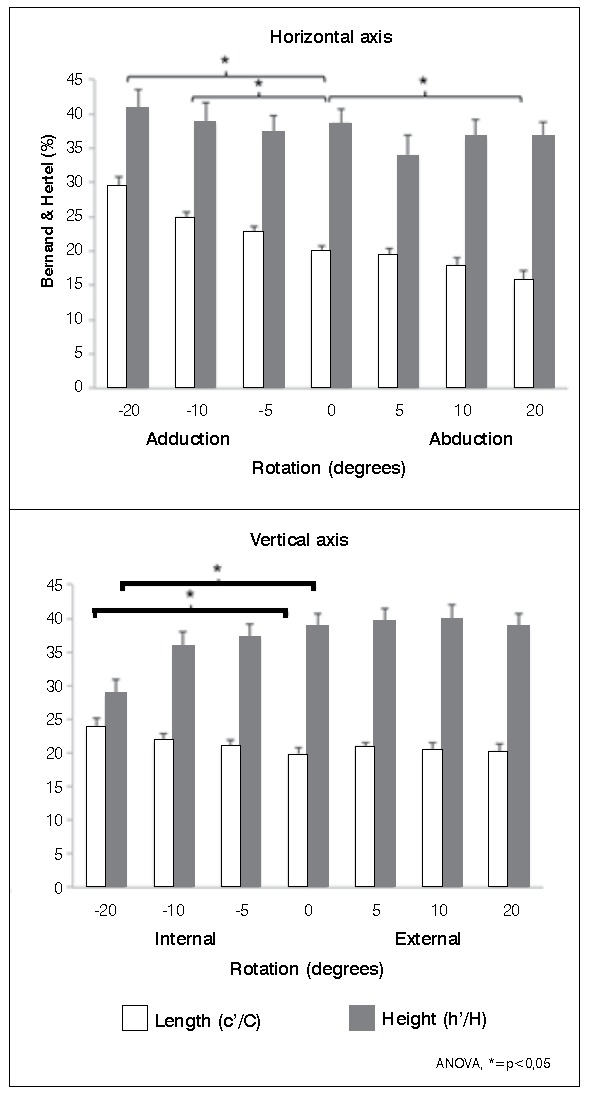
Simulated central femoral ACL tunnel positioning in different horizontal (left) and vertical (right) axes rotation analyzed by Bernard and Hertel method.

F [6,20]=4.06, p=0.001 and F[6,20]=3.45, p=0.003, respectively) for vertical and horizontal axes. (Figure 7)

## Discussion

The clinical relevance of this study is related to methodological accuracy of a standardized transparent 3D CT scan protocol that analyzes anatomical landmarks and ACL footprint. 

Despite well-described rendered 3D CT scan measurement protocols,^14^ this study points out some interesting methodological issues that may contribute to accuracy of ACL tunnel position measurement.

To determine the effect of tunnel position on biomechanical studies or functional outcomes, it is mandatory to have a precise and reproducible measurement tool . Van Eck *et al.* 16 in their anatomic systematic review also demonstrated a methodological concern about misleading ACL reconstruction. 

The most important finding was that this standardized transparent 3D CT scan protocol has high ICC reliability and that small rotation degrees (5^o)^ close to sagittal view^9^^,^ 13 do not alter ACL measurement values. Arthroscopic view, radiographic and 2-dimensional CT or MRI measurements are susceptible to biplanar errors and are not reliable.^9^ Reproducible and accurate imaging protocols are necessary to decrease methodological bias.

Volume-rendering 3D CT scan allows reconstruction of bony landmarks about the knee, as lateral intercondylar ridge and condyle wall contour.^2^^,^ 12 ^,^ 14 However, some anatomical landmarks are superposition images^4^^,^ 15 and many measurement protocols depend upon them (Blumensaat line and condyle wall contour) to define the ACL femoral tunnel insertion site in a sagittal view.^2^^,^ 9 ^,^ 12 ^,^ 14 This issue is technically important because the relationship between ACL femoral tunnel positioning and anatomical landmarks is not linear. 

Forsythe *et al.* 14 and Lertwanich *et al.* 15 recommended that Blumensaat line could be substituted by the most anterior edge of the femoral notch roof or highest point of the anterior aperture of the intercondylar notch, as Blumensaat line does not appear on a conventional rendered 3D CT scan. 

This standardized transparent 3D CT protocol uses the entire intercondylar roof to calculate Blumensaat line. We suggest that Blumensaat line could be used as a reference line in 3D CT scan with transparent properties presented in some 3D reconstruction softwares without accuracy compromise. Inoue *et al.* 9 also showed that this transparent processing image may be useful to confirm surgical techniques.

To our knowledge, most 3D CT scan protocols align posterior femoral condyles wall.^14^ We also considered an inferior condyle wall alignment, as *"varus"* and *"valgus"* (horizontal plane rotation) may alter the neutral sagittal view.^9^^,^ 13 Van Eck *et al.* 17 also showed the importance of *"varus"* and *"valgus"* alignment in a plain radiograph model. More than 100 *"valgus"* could introduce significant error in tunnel position estimation.^17^ We believe that both inferior and posterior femoral walls alignment and the presence of entire inter-condyle roof can standardize 3D CT Blumensaat's line angle and Bernard and Hertel quadrant.

The quadrant method proposed by Bernard and Hertel^12^ is one of the most commonly used techniques to define the insertion point of the ACL on a true lateral image.^14^ It was originally described for radiographies, but is also utilized in 3D CT scan evaluation.^2^^,^ 10 ^,^ 11 ^,^ 14 ^,^ 15 In rendered and cropped 3D CT scans, high levels of intra- and inter-observer reliability were demonstrated^14^^,^ 15 ^,^ 17 for this quadrant method technique, as the present study demonstrated.

Transparent 3D CT method described by Inoue *et al.* 9 has some limitations showed by own authors, that are related with condyle size and small femoral shaft obtained in conventional CT scans that affects the coordinate measurements. For this reason, we believe Bernard and Hertel^12^ method is more appropriate.

Piefer *et al.* 18 showed in their anatomical and radiological systematic review of literature that center of ACL footprint was located at 28.5% (23.5% - 43.1%) of length and 35.2% (27.5% - 44.2%) of height related to Blumensaat line. Our simulated central ACL tunnel measures were quite similar to these authors, as demonstrated in Figure 4.^2^^,^ 11 As argued by Hofbauer *et al.* 19 and Desai *et al.*,^20^ the mainstay of treatment is surgical management with an emphasis on restoring native anatomy.

The main limitation of this study is related to arbitrary choice of central ACL tunnel positioning (below lateral intercondylar ridge and in the middle of ACL footprint). ACL footprint cannot be seen as clearly as in MRI studies and it was not directly measured by anatomical dissection. However, this is a limitation for calculating the exact value of ACL height (h'/H) and length (c'/C), but not for validating ICC transparent 3D CT scan imaging technique or analyzing a mathematical model of central ACL tunnel position rotations, which were the major topics of this study.

Future studies are suggested to compare this new transparent CT image technique of Bernard *et al.* 12 method and the conventional image-rendering and cropped 3D CT scan protocol.

## CONCLUSION

This new transparent 3D-CT protocol is an accurate and reproducible method that can be applied for ACL femoral tunnel or footprint measurement with high ICC reliability.
